# A post-transplant infection by *Nocardia cyriacigeorgica*

**DOI:** 10.1099/acmi.0.000569.v3

**Published:** 2023-11-08

**Authors:** Nageswari Gandham, Sriram Kannuri, Aryan Gupta, Sahjid Mukhida, Nikunja Das, Shahzad Mirza

**Affiliations:** ^1^​ Department of Microbiology, Dr. D. Y. Patil Medical College, Hospital and Research Centre, Dr. D. Y. Patil Vidyapeeth University, Pune, Maharashtra, India

**Keywords:** *Nocardia cyriacigeorgica*, nocardiosis, opportunistic infection, renal transplantation

## Abstract

Nocardia are Gram-positive, acid-fast, filamentous bacteria that cause opportunistic infections in susceptible populations. We describe a case of post-transplant infection of pulmonary nocardiosis caused by the rare strain *Nocardia cyriacigeorgica* and the challenges faced in reaching a definitive diagnosis. This case report emphasizes on keeping nocardiosis as a differential diagnosis in transplant recipients, as this disease is largely underdiagnosed and underreported.

## Data Summary

No data was generated or reused.

## Introduction

In patients receiving immunosuppressive therapy after renal transplantation, the possibility of opportunistic infections increases several fold. One such life-threatening opportunistic infection is nocardiosis [1], caused by organisms that are mainly inhabitants of water and soil [2]. Among the various species causing the said disease, *Nocardia asteroides* complex is most common, followed by *Nocardia brasiliensis and Nocardia otitidiscaviarum* [3, 4].

Looking at the spectrum of diseases caused by *Nocardia *, it most often involves the respiratory system, but it also shows affinity towards the brain, skin and soft tissues [5]. Although it rarely affects immunocompetent patients, a positive case is most likely a result of inoculation of the organism directly through foreign object penetration into the body [4]. Incidence of nocardiosis after solid organ transplant is rare in comparison to other opportunistic infections, amounting to only ~0.6 % [6]. T-cell depleting therapies, including antithymocyte globulin, increase the risk for opportunistic infection. Infected patients mostly present with signs and symptoms of lower respiratory tract infections that do not respond to routine empirical antibiotics, along with progressive deterioration in the general condition of the patient [2]. Microbiological diagnosis of such cases can be tricky given the paucity of organisms in respiratory samples after ruling out other common causes of the infections such as tuberculosis, bacterial pneumonia and invasive fungal disease. Traditional techniques along with recent advancements in microbiology can be combined to reach an accurate diagnosis in such cases [7]. Here, we highlight a case of pulmonary nocardiosis in an immunocompromised patient post-renal transplantation caused by a rare species, *Nocardia cyriacigeorgica *.

## Case presentation

**Fig. 1.**
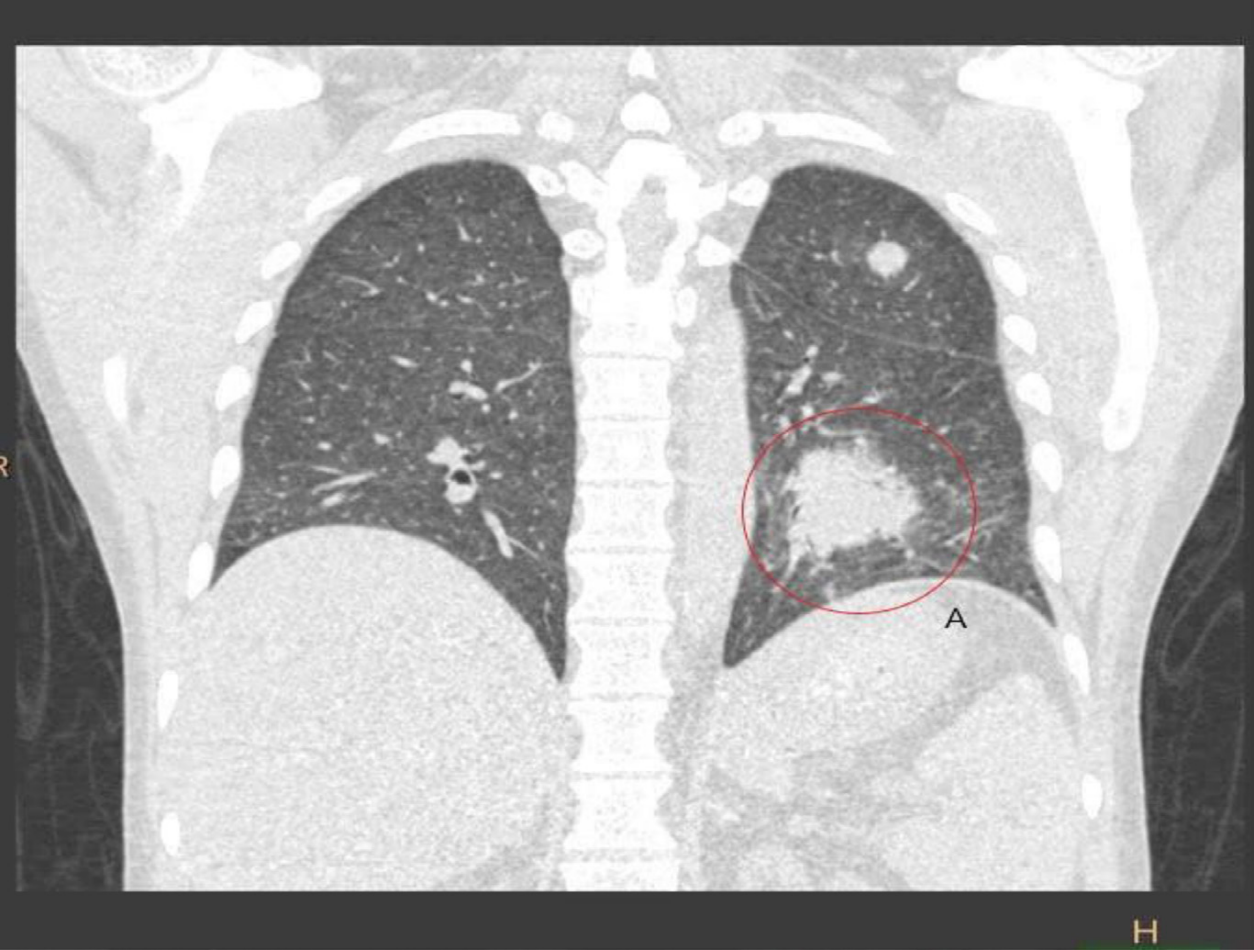
Coronal section of computed tomography scan showing multiple nodules and mass-like consolidations in the left lung. A encircles the largest consolidation.

**Fig. 2.**
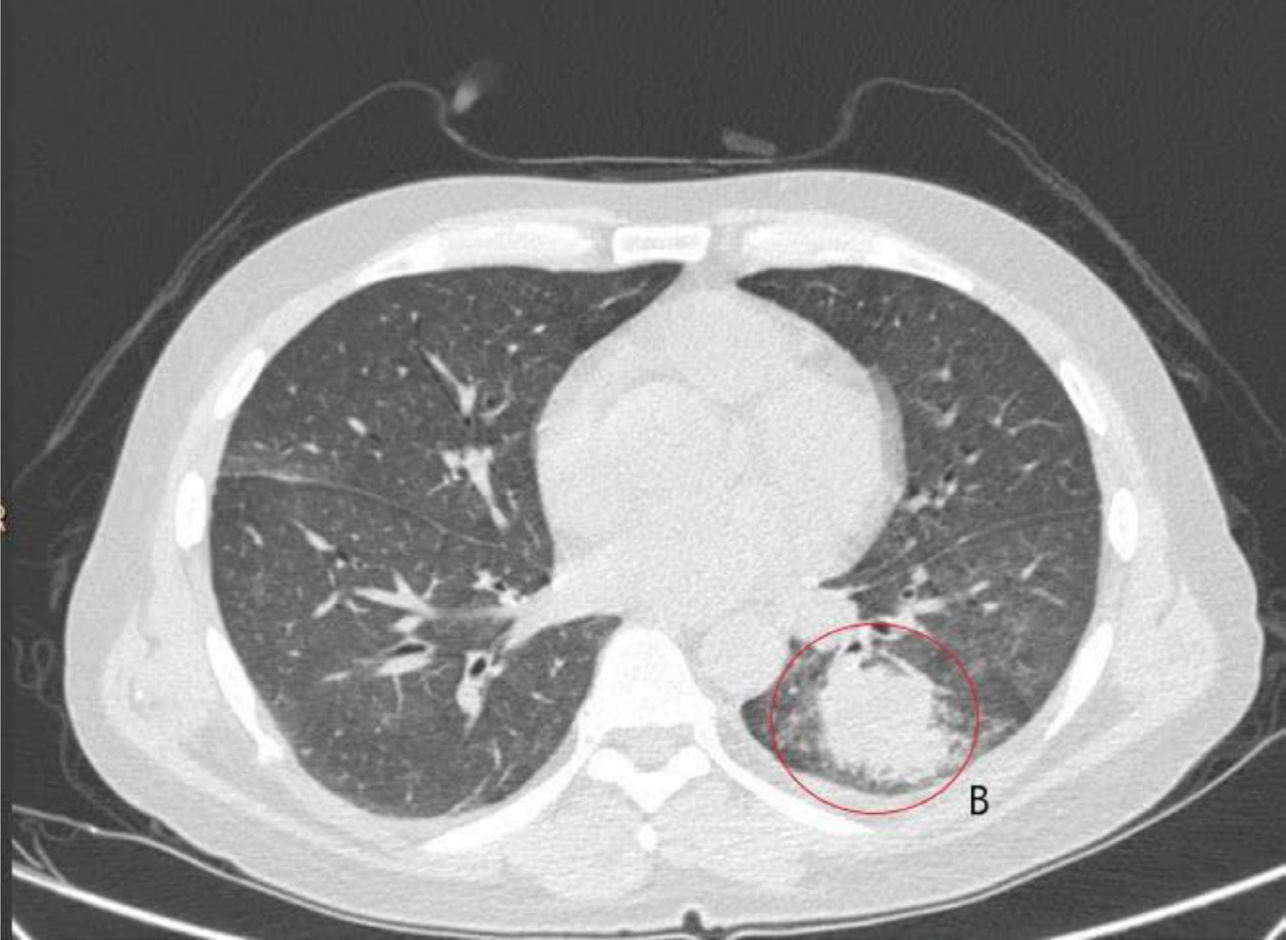
Axial section of computed tomography scan. B encircles the largest consolidation in the left lung.

**Fig. 3.**
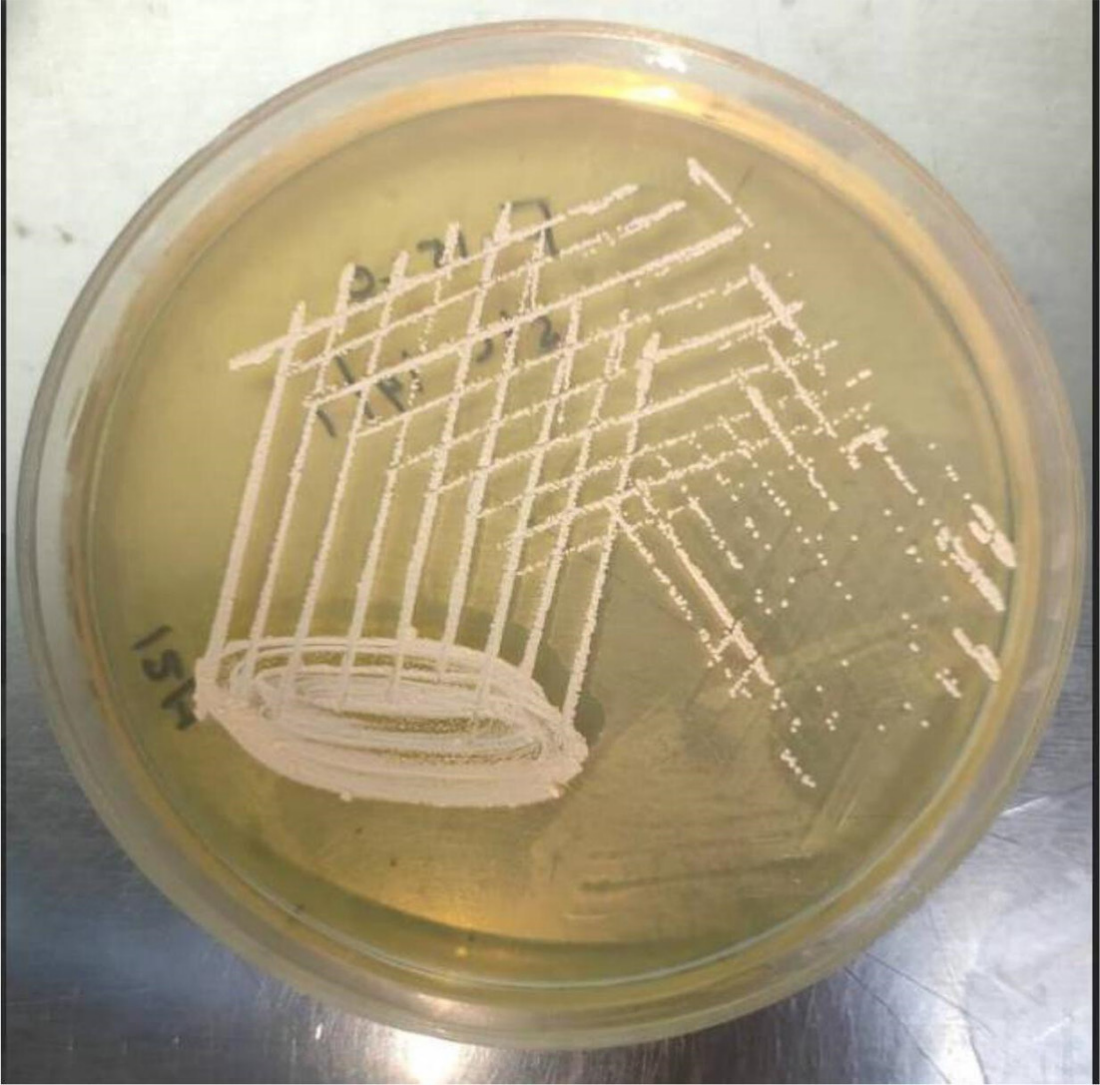
Growth of colonies of the isolate on Mueller–Hinton agar.

**Fig. 4.**
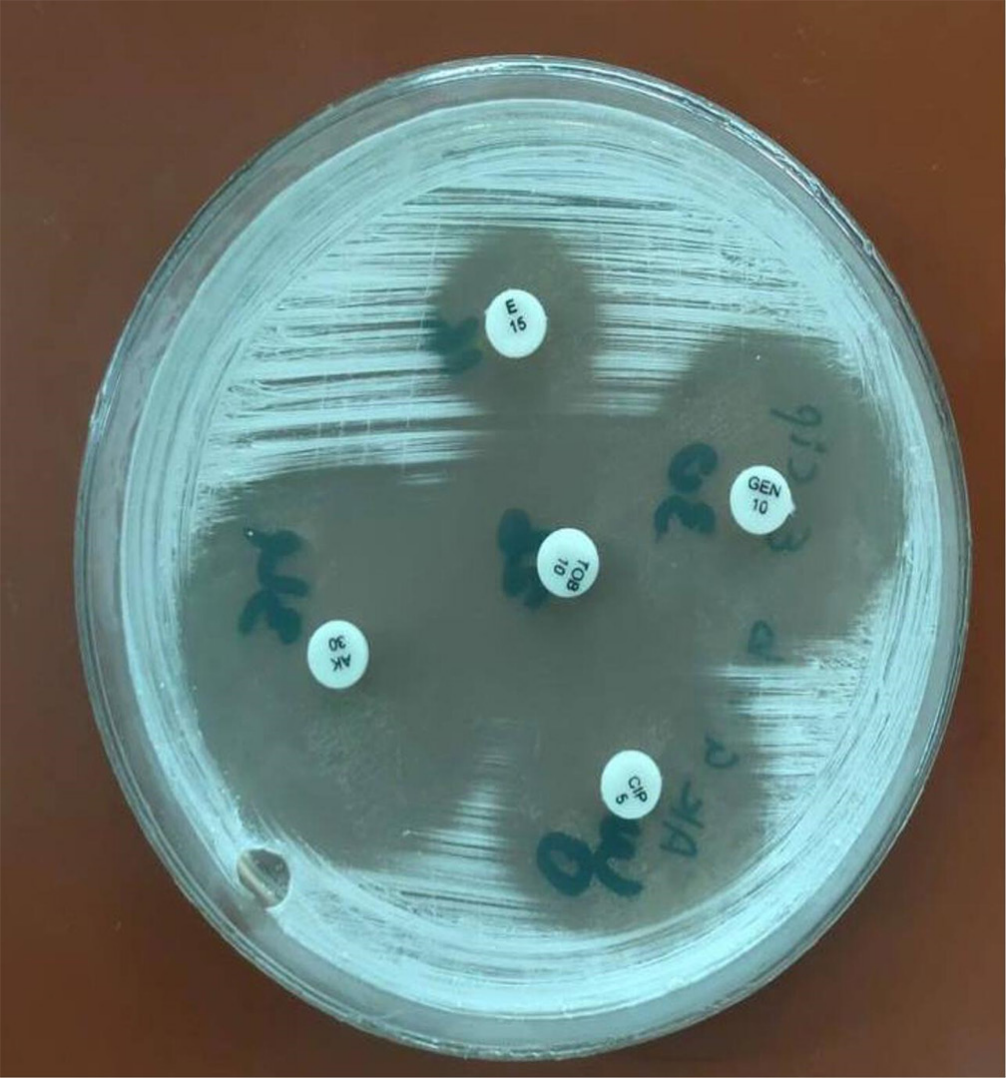
*
Nocardia* spp. susceptibility to amikacin (Ak), gentamicin (Gen), tobramycin (Tob) and ciprofloxacin (Cip).

A 41-year-old male with a history of grade 5 chronic kidney disease, diagnosed 2 years ago, had an HLA 6/6 mismatch living-related renal transplant: wife to husband in the month of April 2022. Post-transplant, the patient was put on immunosuppressive medications. Approximately 2 months after the transplant, the patient gradually developed fever, which was intermittent in nature, and a cough with expectoration, which exacerbated on supine position. To evaluate the aetiology, sputum and broncho-alveolar lavage (BAL) sample collection was done, and the specimens were sent for microbiological investigation. Chest X-ray, ultrasonography of the thorax and high-resolution computed tomography (HRCT) revealed bilateral nodular consolidations that were progressive in nature (Figs 1 and 2). The patient’s sputum and BAL culture was negative for bacteria, fungi and *Mycobacterium tuberculosis *. As a last resort, a closed transbronchial lung biopsy was done given the insignificant sputum and BAL studies, and progressive deterioration in the condition of the patient. A Gram-stain smear of excised tissue exhibited a Gram-positive branching filamentous structure, and Ziehl–Neelsen (ZN) stain of the same revealed a weak acid-fast branching filamentous structure. A modified acid-fast staining using 0.5–1 % sulphuric acid as a decolourizer (rather than the conventional 20 % sulphuric acid) serves as an extremely useful guide for the diagnosis of *Nocardia *, aligning with the observations of Gram-stain and ZN stain. The modified ZN revealed acid-fast branching filamentous bacilli. The inoculation of the tissue sample was done on blood, chocolate and MacConkey’s agar. On blood and chocolate agar, dry, chalky white, medium-sized colonies were observed, whereas no colonies were observed on MacConkey’s agar. To observe the gross appearance in liquid media, tissue was also inoculated into Robertson’s cooked meat (RCM) broth, in which large, chalky white pellicles were found. Smears from the colonies of both blood and chocolate agar were subjected to Gram, ZN and 1 % modified ZN staining, and subsequent microscopy revealed a Gram-positive, acid-fast, branching, filamentous bacilli. The colonies were also inoculated onto Mueller–Hinton agar by lawn technique to check for drug susceptibility, as a method to determine the possible group of *Nocardia* causing the condition (Fig. 3). Drugs used for differentiation by susceptibility were tobramycin, amikacin, gentamicin and erythromycin [7]. The cultured isolate was found to be susceptible to tobramycin, amikacin and gentamicin, but was resistant to erythromycin (Fig. 4). Based on this pattern of susceptibility, it was suspected that the organism was among the following, i.e. *N. asteroides* I/VI, *N. brasiliensis *, *Nocardia pseudobrasiliensis* and *N. otitidiscaviarum *. For complete identification of the species, a MALDI-TOF (matrix-assisted laser desorption/ionization-time of flight) MS ID test (MALDI Biotyper Sirius; Bruker) was performed on the sample. The isolate was identified as *N. cyriacigeorgica *. The patient was started on injection meropenem 1 g, twice daily for 10 days, and oral co-trimoxazole once daily (sulfamethoxazole 800 mg/trimethoprim 160 mg) for 25 days, and further was put on prophylaxis with oral co-trimoxazole – 200 μg for 3 weeks. The patient’s response to treatment was favourable. Follow-up for the patient was uneventful and subsequently a lung biopsy was done for confirmation (as the patient was immunocompromised and to avoid further complications), which revealed no similar bacterial culture.

## Discussion

*Nocardia* species are known to be Gram-positive, filamentous, branching bacilli that are also partially acid-fast. The prevalence of nocardiosis is rare in comparison to other bacterial infections; however, under certain conditions, it should be kept as a differential diagnosis that should not be ignored [4]. The incidence of *Nocardia* infections after a solid organ transplant can be between 0.04 and 3.5 % [8], and the incidence in renal transplant patients can be between 0.04 and 1.2 % [9]. According to a case–control study conducted by Peleg *et al*., the risk factors for nocardiosis in solid organ transplant recipients can be attributed to high-dose steroid therapy, infection with cytomegalovirus (CMV) and high median calcineurin inhibitor levels in the preceding 30 days (>300 ng ml^−1^ for cyclosporine and >15 μg ml^−1^ for tacrolimus) [6]. It was also noted that patients receiving tacrolimus were at a higher risk of contracting nocardiosis than those receiving cyclosporine [10]. Our patient also had tacrolimus trough levels of 6 μg ml^−1^ and high-dose corticosteroids to avoid organ rejection due to HLA mismatch. It is reported that *Nocardia* infections usually present within a period of 6 months and rarely 1 year post-transplant, similarly our patient presented with the typical symptoms of cough, fever and breathlessness [3]. Due to the lack of distinctive clinical findings or radiological evidence indicative of a *Nocardia* infection, Gram-staining of sputum samples remains a vital tool for diagnosis [3, 5, 7]. Bilgarnia *et al*. and Flohr *et al*. reported two other cases of pulmonary nocardiosis. Both cases underwent re-transplantation, and preconditioning was done using plasmapheresis and rituximab. Post-transplant, these patients were maintained on mycophenolate mofetil, tacrolimus and steroids [11, 12]. We received multiple sputum specimens, but all their Gram-stain reports were insignificant and ZN staining was negative for the presence of acid-fast bacilli. For better diagnostic sensitivity, BAL samples were then taken, which proved to be a failure. As prior investigations proved to be inconclusive, more invasive techniques were indicated [13]. Invasive samples like BAL were sent, which were negative for infections like mycobacterium, but because of the persistent radiological evidence, a lung biopsy was done that proved *Nocardia* species, an opportunistic infection. The lung biopsy samples assisted in isolating the causative organism by providing the above-mentioned picture in staining and culture. Antibiotic susceptibility for *Nocardia* can be identified by broth microdilution [14], or by the Kirby–Bauer disc diffusion method [15] (Table 1). In our study, broth microdilution was not done due to technical reasons. An accurate diagnosis was made because of the cultures; after which, treatment was initiated promptly to deal with infection. MALDI-TOF MS test, wherever applicable, can be used to diagnose such cases, as it gives fast and reliable results for such difficult-to-diagnose pathogens [16]. Prophylactic treatment for *Nocardia* usually involves long-term co-trimoxazole. Alternatively, drugs such as amikacin, imipenem, meropenem and third-generation cephalosporins have been shown to have efficacy against *Nocardia in vitro* [17].

**Table 1.** Various case reports dealing with *Nocardia* species isolation and their findings

No.	Authors	Brief description of patient/illness	Species isolated	Treatment	Antibiotic-susceptibility pattern by Kirby–Bauer disc diffusion method	Antibiotic-susceptibility pattern by broth microdilution method
1	Khadka & Shah [15]	Cutaneous nocardiosis post-renal transplant	* Nocardia asteroides *	Trimethoprim/ sulfamethoxazole	Yes.	No
2	Zintgraff *et al*. [14]	Brain abscess in an immunocompromised patient	* Nocardia farcinica *	Trimethoprim/ sulfamethoxazole and linezolid	No	Yes
3	Rodríguez-Lozano *et al*. [18]	Post-traumatic endophthalmitis	* Nocardia nova *	Amikacin, imipenem	Yes (by Etest strips; bioMèrieux)	No
4	Kuchibiro *et al*. [19]	Pulmonary nocardiosis	* Nocardia mexicana *	Trimethoprim/ sulfamethoxazole	No	Yes
5	Shokri *et al*. [20]	Pulmonary and cutaneous nocardiosis	* Nocardia mexicana *	Linezolid/amikacin	No	Yes

### Conclusion

Amongst a plethora of opportunistic infections affecting immunocompromised patients, *Nocardia* should be kept as a differential diagnosis in renal transplant recipients presenting with a pulmonary infection. Invasive diagnostics can help the patient out with an early diagnosis of nocardiosis and prevent disease dissemination. If needed, the use of advanced techniques such as MALDI-TOF MS should be sought for an early and reliable diagnosis.
